# Major Surface Glycoproteins of Insect Forms of *Trypanosoma brucei* Are Not Essential for Cyclical Transmission by Tsetse

**DOI:** 10.1371/journal.pone.0004493

**Published:** 2009-02-18

**Authors:** Erik Vassella, Michael Oberle, Simon Urwyler, Christina Kunz Renggli, Erwin Studer, Andrew Hemphill, Cristina Fragoso, Peter Bütikofer, Reto Brun, Isabel Roditi

**Affiliations:** 1 Institut für Zellbiologie, Universität Bern, Bern, Switzerland; 2 Swiss Tropical Institute, Basel, Switzerland; 3 Institut für Parasitologie, Universität Bern, Bern, Switzerland; 4 Institut für Biochemie und Molekulare Medizin, Universität Bern, Bern, Switzerland; Swiss Tropical Institute, Switzerland

## Abstract

Procyclic forms of *Trypanosoma brucei* reside in the midgut of tsetse flies where they are covered by several million copies of glycosylphosphatidylinositol-anchored proteins known as procyclins. It has been proposed that procyclins protect parasites against proteases and/or participate in tropism, directing them from the midgut to the salivary glands. There are four different procyclin genes, each subject to elaborate levels of regulation. To determine if procyclins are essential for survival and transmission of *T. brucei*, all four genes were deleted and parasite fitness was compared in vitro and in vivo. When co-cultured in vitro, the null mutant and wild type trypanosomes (tagged with cyan fluorescent protein) maintained a near-constant equilibrium. In contrast, when flies were infected with the same mixture, the null mutant was rapidly overgrown in the midgut, reflecting a reduction in fitness in vivo. Although the null mutant is patently defective in competition with procyclin-positive parasites, on its own it can complete the life cycle and generate infectious metacyclic forms. The procyclic form of *T. brucei* thus differs strikingly from the bloodstream form, which does not tolerate any perturbation of its variant surface glycoprotein coat, and from other parasites such as *Plasmodium berghei*, which requires the circumsporozoite protein for successful transmission to a new host.

## Introduction

Two diseases that are prevalent in sub-Saharan Africa, human sleeping sickness and Nagana, a disease of domestic animals, are caused by African trypanosomes. The spread of these diseases is determined by tsetse flies (*Glossina* spp.) capable of transmitting trypanosomes from one mammalian host to the next [1]. Successful transmission of *Trypanosoma brucei* first involves the establishment of an infection in the insect midgut. This is followed by a maturation phase in which the parasites migrate to the salivary glands and finally give rise to metacyclic forms that can be transmitted to a new mammalian host.

Unlike many other unicellular parasites, *T. brucei* remains extracellular throughout its life cycle and survives in the circulation of the mammalian host and the protease-rich environment of the fly midgut by expressing specific coats of glycosylphosphatidylinositol (GPI)-anchored surface glycoproteins [2]–[4]. In the mammalian host, bloodstream forms of the parasite are covered by a coat consisting of variant surface glycoprotein (VSG). The VSG coat acts as a physical barrier that prevents access to underlying molecules and destruction of the parasite, at the cost of provoking a humoral immune response against itself. The process of antigenic variation, involving the periodic recruitment and expression of a new VSG gene from a repertoire of several hundred genes, prevents lysis by antibodies directed against previous coats. An intact VSG coat appears to be essential for the parasite, both in vivo and in culture, since perturbation of GPI anchor synthesis [5] or down-regulation of the VSG mRNA by RNA interference [6] is lethal.

When a tsetse fly feeds on an infected host, bloodstream forms are exposed to a multitude of proteases in the insect's midgut. Certain proteases, such as trypsin, kill slender (proliferating) bloodstream forms, but promote the differentiation of stumpy (cell-cycle arrested) bloodstream forms to the next stage of the life cycle, the procyclic form [7]. Synchronous differentiation of the stumpy form to the procyclic form can be induced in culture, either by proteases [7] or, more commonly, by the addition of citrate or *cis*-aconitate to the medium [8], [9]. As the trypanosome differentiates, a membrane-anchored metalloprotease (MSP B) cleaves VSG from the surface, making way for a new stage-specific coat of several million copies of procyclins within the space of a few hours [10], [11]. The two classes of procyclins, EP and GPEET, are characterised by internal dipeptide and pentapeptide repeats, respectively. These repeats are resistant to cleavage by MSP-B and tsetse midgut proteases, although the procyclin N-termini are removed by the latter [12], [13], for reasons that are unclear [14]. Expression of different forms of procyclin is regulated temporally. A few hours after differentiation is induced, trypanosomes express all three EP isoforms (EP1, EP2, EP3) and GPEET [15]. This is followed by a surge of GPEET synthesis, making it the predominant component of the coat of early procyclic forms [12], [15]. In vivo, GPEET is repressed after a few days and replaced by N-glycosylated forms of EP, EP1 and EP3, in late procyclic forms [12]. In culture, trypanosomes can be kept as early procyclic forms when glycerol is present in the medium and induced to differentiate to late procyclic forms when glycerol is removed [16]. EP procyclins are also expressed by the mesocyclic form in the anterior midgut and by trypomastigotes in the proventriculus [17], but not by the epimastigote form that colonises the salivary glands or the metacyclic form [18]. Epimastigote forms express a family of GPI-anchored proteins known as *brucei* alanine-rich proteins (BARPs) [4] while metacyclic forms are covered by VSG [19].

In contrast to the VSG coat of bloodstream forms, procyclins are not essential for procyclic culture forms. We previously obtained an EP/GPEET null mutant by deleting the genes from the bloodstream form and then triggering the knockout to differentiate in culture [20]. After shedding the VSG coat, the null mutant required almost two months before it was able to proliferate normally. Analysis of this mutant revealed that, in the absence of procyclin polypeptide precursors, free GPI anchors were on the surface, forming a glycocalyx. Since both the wild type parental strain and the mutant were very poorly infectious for tsetse, we were unable to draw any conclusions about the function of procyclins in vivo. Several mutants have been generated in procyclic forms and tested in tsetse, but none of these gives a definitive answer about the role of procyclins in the fly. One mutant (Nour 6C) that lacked all EP genes, but still retained a single GPEET gene, was almost an order of magnitude less efficient than the wild type at establishing heavy midgut infections in flies [21]. The phenotype was partially restored by reintroduction of either EP1 or EP2, suggesting that they play a protective role. Mutants defective in GPI anchor biosynthesis showed different phenotypes. Mutants lacking the GPI transamidase (encoded by *GPI8*) [22], or GlcNAc-phosphatidylinositol de-N-acetylase (encoded by *GPI12*), [23] no longer synthesised membrane-bound EP or GPEET and were also extremely inefficient at establishing midgut infections. This could be due to a lack of GPI-anchored proteins other than procyclins, however. In contrast, deletion of the gene encoding another GPI biosynthetic enzyme, *GPI10*, had little effect on the ability of procyclic forms to infect flies [5].

In addition to any functions they might have in the first phase of infection, another possibility is that procyclins are required in order for *T. brucei* to leave the midgut and migrate to the salivary glands. It has not been possible to test this hypothesis previously as none of the mutants described above could complete the life cycle in the fly (a common problem when procyclic forms are cultured for any length of time). In order to study the function of procyclins throughout the life cycle we deleted all procyclin genes from a fly-transmissible strain. A surprising outcome of these experiments was that trypanosomes without a procyclin coat are still transmissible by tsetse, albeit with a substantial reduction in the prevalence and intensity of salivary gland infections.

## Results

### GPEET is not required for the establishment of infection

**Figure 1:**
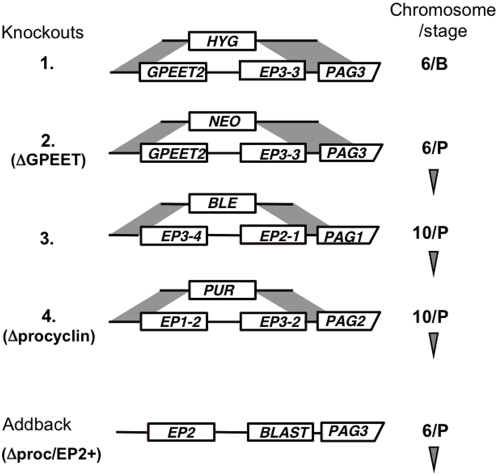
Schematic depiction of the procyclin loci in AnTat 1.1 and lineage of the knockouts [15]. GPEET2 is a variant that has 5 rather than 6 copies of the pentapeptide repeat. Deletion constructs containing antibiotic-resistance genes were designed to delete tandemly linked procyclin genes by homologous recombination (see Materials and Methods). In the addback Δproc/EP2+, the neomycin-resistance gene was replaced by EP2 procyclin and the blasticidin resistance gene. The truncated 3′ UTR (ΔLII) downstream of EP2 ensures high levels of expression in procyclic forms [36], [40]. B: bloodstream form; P: procyclic form. Arrowhead: transmitted through tsetse and mice.

The procyclin mutants described to date [14], [20], [21], [24] were generated in laboratory-adapted stocks that had either been passaged in rodents or maintained in culture for an extended period of time. For unknown reasons, these trypanosomes lose their ability to complete the life cycle in the fly. We therefore decided to generate a new null mutant in a fly-transmissible clone, AnTat 1.1. The basic arrangement of procyclin genes is the same as in *T. brucei* 427 [20]. Both stocks have procyclin loci on chromosomes 6 and 10. In Antat 1.1, the locus on chromosome 6 is homozygous for the gene pair GPEET and EP3-3, while the two EP genes on chromosome 10 are heterozygous (Figure 1). The first knockout of the procyclin locus on chromosome 6 was generated in bloodstream forms. Bloodstream forms of pleomorphic stocks are notoriously difficult to transfect and maintain in culture, however, so subsequent knockouts were generated in procyclic forms. To maintain their competence to complete the life cycle, trypanosome clones were transmitted through tsetse and mice after each round of transfection, and triggered to differentiate to procyclic forms in vitro. Deletion of the procyclin locus from the second copy of chromosome 6 gave rise to the mutant ΔGPEET that lacked the GPEET gene and the adjacent EP3-3 gene (Figure 1).

**Figure 2:**
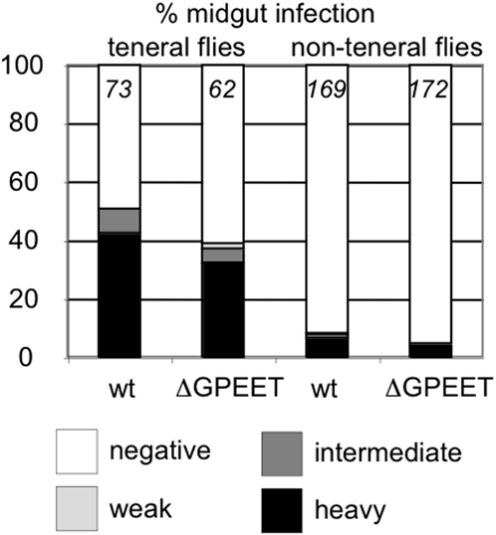
Comparison of the prevalence of midgut infections in tsetse flies exposed to stumpy bloodstream forms of wild type AnTat 1.1 (wt) or ΔGPEET. Infections were classified as described [21]. Teneral flies were infected with trypanosomes in their first blood meal after hatching. Non-teneral flies were infected with the second blood meal when they were 4–6 days old. Infection prevalence and intensity were assessed 11–14 days post infection. The number of flies per experimental group is indicated at the top of each column. Statistical analysis showed no significant differences between the wild type and the mutant in the prevalence or intensities of midgut infections.

Since GPEET is expressed abundantly for the first few days of an infection, and then repressed thereafter [16], we analysed the ability of bloodstream forms of the mutant to establish infections when given to teneral flies as part of their first blood meal. The flies were dissected 11–14 days post infection and graded as described previously [21]. These experiments revealed only slight differences in the prevalence and intensity of midgut infections, with the wild type being able to establish heavy infections in 42.5% of flies and ΔGPEET in 32.3% (Figure 2); these differences were not statistically significant. Non-teneral flies (flies that have taken a blood meal) are more refractory to infection. This is probably a closer reflection of the situation in the wild and might potentiate differences that are not detectable when conditions are optimised for high infection rates in teneral flies. Of the flies exposed to trypanosomes in their second blood meal, ∼7 times fewer flies were able to establish heavy midgut infections, but this applied to both the wild type and ΔGPEET (Figure 2). In the standardised procedure that we use for transmission experiments, the flies normally receive an inoculum of about 4×10^4^ trypanosomes (assuming that they consume ∼20 µl blood), which is considerably more than from an infected animal. However, reducing the inoculum given to teneral flies by a factor of 20 did not reveal any difference between ΔGPEET and the wild type (data not shown).

### A procyclin null mutant is fly-transmissible

**Figure 3:**
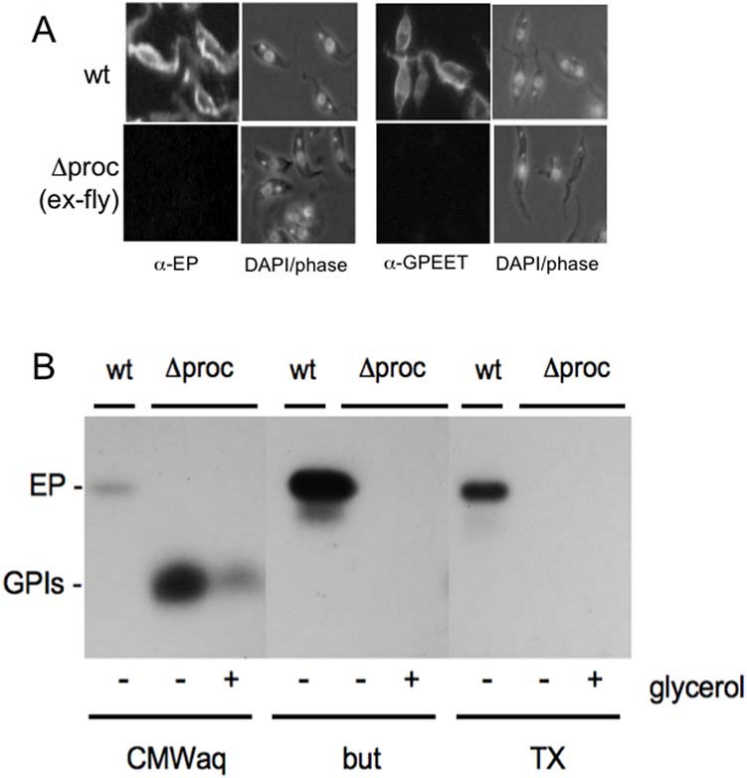
A. Immunofluorescence analysis of procyclic forms of trypanosomes after cyclical transmission through tsetse flies (ex-fly). Procyclic forms of the wild type (wt) and Δprocyclin (Δproc) were labelled with the monoclonal antibody TBRP1/346 against EP or with a rabbit polyclonal antiserum against GPEET. Right panels: DAPI images (dark field or phase) from the same fields. B. Analysis of GPI-anchored molecules. Wild-type trypanosomes (wt) and Δprocyclin (Δproc), cultured as early procyclic forms (+glycerol) or late procyclic forms (−glycerol), were labelled with [^3^H]ethanolamine as described [20]. The delipidated pellets were extracted with CMW to solubilise free GPIs, followed by 9% butan-1-ol (but) and 0.1% Triton X-100 (TX) to solubilise procyclins. The CMW extracts were dried and partitioned between water and butan-1-ol; the aqueous phase contains free GPIs (CMWaq). All fractions were analyzed by SDS-PAGE and fluorography. The positions of EP procyclin (EP) and free GPIs (GPIs) are indicated.

Since no obvious phenotype was observed in trypanosomes lacking GPEET, apart from a slightly lower prevalence of heavy midgut infections, we proceeded to delete the remaining procyclin genes. After two further rounds of transfection many drug-resistant clones were isolated, but only one clone of more than 12 that were examined had deleted both of the remaining two copies of EP (Figure 1 and Supplemental Figure S1). This is reminiscent of our earliest attempts to generate procyclin knockouts, which also suggested that retention of a procyclin gene was advantageous for procyclic forms [21]. Procyclic forms of the null mutant (Δprocyclin clone#6) were used to infect teneral flies. To our astonishment, Δprocyclin was able to complete the life cycle in tsetse and infect mice. The resulting bloodstream forms were triggered to differentiate to procyclic forms and subjected to immunofluorescence analysis with anti-EP and anti-GPEET antibodies (Figure 3A) and Southern blot analysis (Supplemental Figure S1). Both confirmed that the trypanosomes that survived transmission were indeed procyclin null mutants. Procyclic forms were also subjected to [^3^H]ethanolamine-labelling and fractionated according to standard procedures that separate free GPI anchors and GPI-anchored proteins (Figure 3B). Both early procyclic forms and late procyclic forms of Δprocyclin expressed increased amounts of free GPIs, as shown previously for the EP/GPEET null mutant [20], and neither stage expressed any detectable GPI-anchored proteins as alternatives to procyclins (Figure 3B). Unlike the EP/GPEET mutant, which was generated entirely in the bloodstream form [20], Δprocyclin did not show defects in differentiation from the bloodstream to the procyclic form, nor did it undergo growth arrest shortly after differentiation (data not shown).

**Figure 4:**
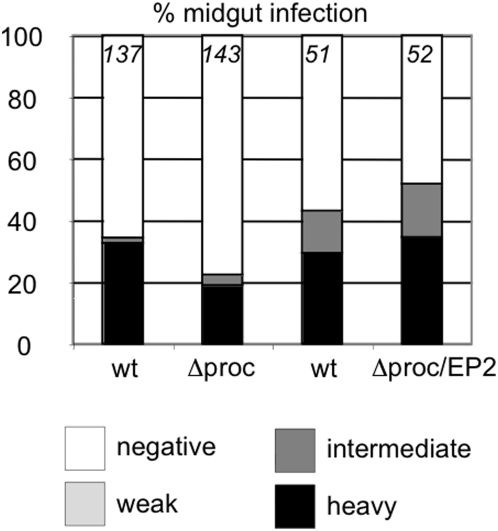
Comparison of midgut infection rates in flies infected with stumpy bloodstream forms of wild type AnTat 1.1 (wt), Δprocyclin (Δproc) or the addback mutant Δproc/EP2+. Midgut infection rates were determined 11–14 days post infection and graded as described in the legend to Figure 2. The number of flies per experimental group is indicated at the top of each column.

To obtain quantitative data on the efficiency of transmission, teneral flies were infected with stumpy bloodstream forms of the wild type or Δprocyclin and monitored for the establishment of midgut and salivary gland infections (Figure 4). Surprisingly, after 11–14 days 18.9% of flies infected with Δprocyclin exhibited heavy midgut infections, compared to 32.8% of flies infected with the wild type (Figure 4; p = 0.01). This is in striking contrast to the EP null mutant Nour 6C which was 5–10 times less efficient at establishing heavy infections than its wild type parent, despite expressing high levels of GPEET [21]. We have previously shown that overexpression of either EP1 or EP2 by the Nour 6C mutant promotes its survival in the midgut [21]. To investigate whether procyclins can rescue the phenotype of Δprocyclin, an addback (Δproc/EP2+) was constructed by integrating an EP2 coding region into the procyclin locus on chromosome 6. When stumpy bloodstream forms of Δproc/EP2+ were used to infect flies, they established heavy midgut infections at the same rate as the wild type (Figure 4).

### Fitness of the mutants in competition experiments

**Figure 5:**
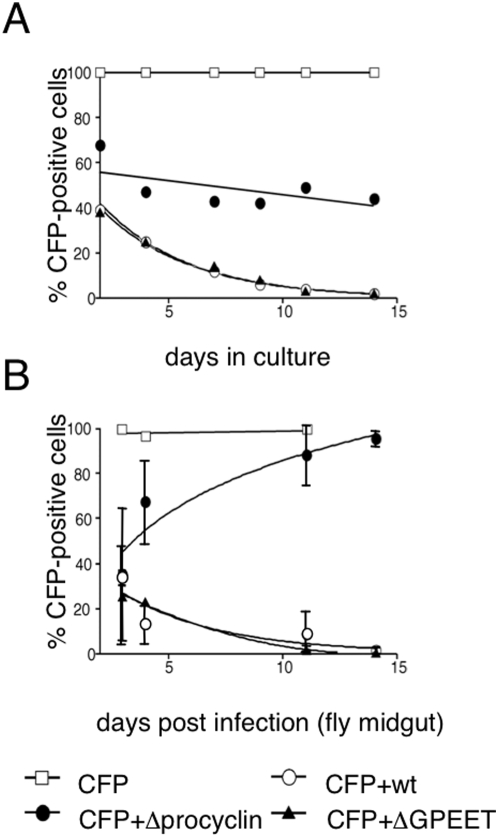
A. Competition with CFP-tagged trypanosomes in culture. Stumpy forms of wild type AnTat 1.1 (wt), ΔGPEET or Δprocyclin (Δproc) were mixed with an equal number of stumpy forms of CFP#5, which expresses cyan fluorescence protein from the procyclin promoter. The mixed cultures were triggered to differentiate by the addition of *cis*-aconitate and incubated at 27°C in SDM-79 supplemented with 10% FBS and 20 mM glycerol. The percentage of CFP-positive cells was determined by observing the cells under a fluorescence microscope. B. Mixed infections with CFP#5. Stumpy forms of the cell mixtures described above were used to infect tsetse flies and the percentage of CFP-positive cells was determined from cells isolated from the midgut of infected flies. As a control for CFP expression, a pure culture of CFP#5 was analysed in parallel.

The experiments described above clearly show that, even without procyclins, trypanosomes can establish midgut infections reasonably successfully. In the long term, however, variants with even a slight competitive disadvantage would be lost from the population. In order to gauge the influence of procyclin genes on fitness, a tagged form of AnTat 1.1 expressing cyan fluorescent protein (CFP#5) was used as a reference in competition experiments [25]. Stumpy bloodstream forms of CFP#5 were mixed with equal numbers of wild type AnTat 1.1, ΔGPEET or Δprocyclin and induced to differentiate by the addition of *cis*-aconitate. The percentage of CFP-positive cells in each culture was monitored over a period of two weeks (Figure 5A). A control culture containing CFP#5 alone became 100% positive within two days of triggering differentiation and remained so throughout the experiment. When CFP#5 was combined with Δprocyclin, the ratio of the two cell types remained reasonably constant over 14 days. In contrast, CFP#5 was overgrown by the wild type and ΔGPEET with the same kinetics and was barely detectable at the last time point. These experiments indicate that Δprocyclin has a similar level of fitness to CFP#5 in culture, but both are less robust than the wild type and ΔGPEET.

In parallel, the same mixtures of bloodstream forms were used to infect teneral flies and midgut infections were analysed (Figure 5B). Wild type AnTat 1.1 and ΔGPEET behaved in the same way as they did in culture, progressively overgrowing CFP#5 over a period of two weeks. Thus there is no indication that ΔGPEET was any less fit than wild type AnTat 1.1 *in vivo*. In contrast to the situation in culture, however, Δprocyclin was unable to maintain its equilibrium with CFP#5 in the fly and was competed out within one cycle of infection.

Experiments were also performed with the addback Δproc/EP2+, which was fitter than Δprocyclin at the beginning of fly infection, but overgrown by CFP#5 by day 7 (data not shown).

### Reduced salivary gland infections and adherence to epithelial cells

**Figure 6:**
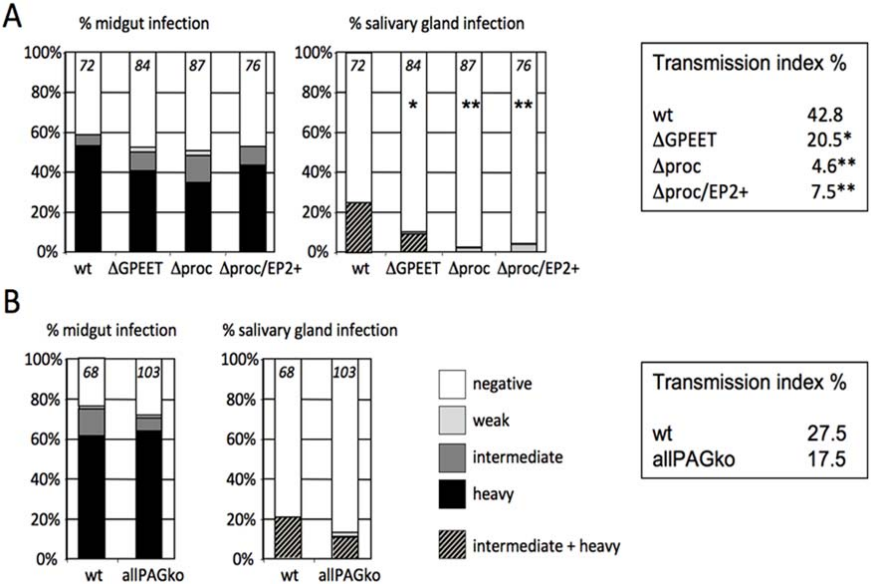
Maturation rates and transmission indices. A. Teneral flies were infected with stumpy bloodstream forms of wild type AnTat 1.1, ΔGPEET, Δprocyclin or Δproc/EP2+. Flies were dissected 28–35 days post infection and graded for the prevalence and intensity of midgut and salivary gland infections. Significant differences between the prevalence and transmission index obtained with wild type AnTat1.1 and various mutants are indicated by asterisks; p<0.05 (*) and p<0.001 (**). No significant differences were observed when the mutants were compared to each other. B. Multiple resistance genes do not reduce the intensity of salivary gland infections. Teneral flies were infected with early procyclic forms of AnTat 1.1 or allPAGko clone 1 [25], which carries the same resistance genes as Δprocyclin. Midgut and salivary gland infections were categorised as described above. The number of flies per experimental group is indicated.

**Figure 7:**
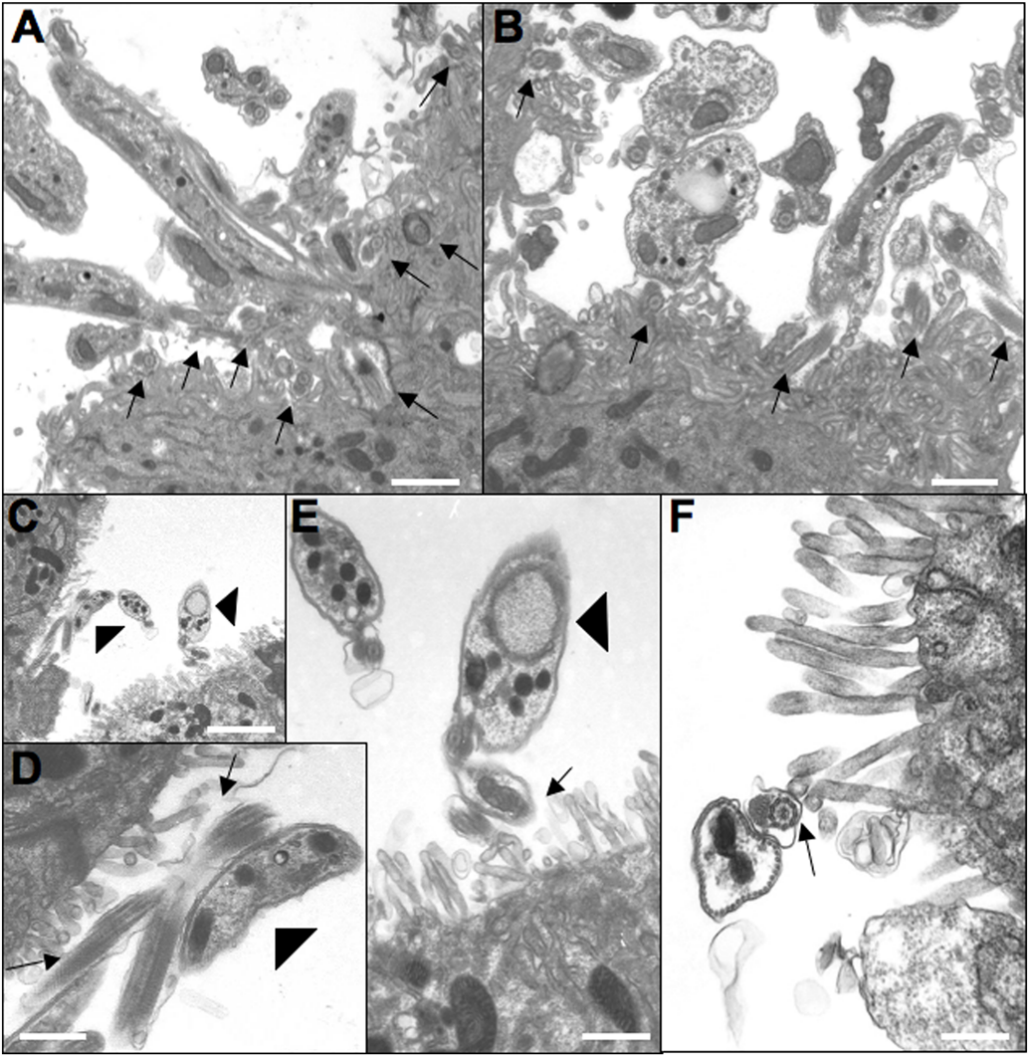
Representative images from sections of salivary glands. (A, B) Salivary glands isolated from flies infected with wild type AnTat1.1. Note that the flagella (marked with arrows) are in very close contact with the epithelial cells and often appear completely entangled with surface microvilli of the salivary gland. (C–F) Salivary glands from flies infected with Δprocyclin. (D) and (E) are higher magnification views of those areas in C marked by arrowheads. Arrows mark the flagella adhering to the epithelia. Note the much lower density of parasites, and the much less pronounced interaction of Δprocyclin with the epithelium compared to wild type AnTat1.1. Bars in (A) = 1.1 µm; (B) = 0.8 µm; (C) = 2.4 µm; (D) = 0.6 µm; (E) = 0.6 µm; (F) = 0.44 µm.

The initial transmission experiments performed to obtain bloodstream forms of each mutant indicated that procyclins were not required for maturation, but gave no information on the efficiency of this process. To study this more precisely, teneral flies were infected with bloodstream forms of wild type AnTat1.1, ΔGPEET, Δprocyclin or Δproc/EP2+. Several independent experiments were performed, with similar results. In the experiment depicted in Figure 6A, both midgut and salivary gland infections were assessed 28–35 days after infection, enabling us to determine the transmission index (percentage of flies with midgut infections also giving rise to mature salivary gland infections). Under the conditions used, wild type AnTat 1.1 produces salivary gland infections in a high proportion of infected flies (25% in this experiment, with a transmission index (TI) of 42.8%). In general, the glands were heavily infected and electron micrographs showed the flagella of epimastigote forms in tight association with the salivary gland epithelia (Figure 7A, B) as has been documented previously [26]. ΔGPEET produced salivary gland infections in fewer flies (10.7% infection, TI = 20.5%); the difference to the wild type was slightly significant statistically (p = 0.021). In contrast, Δprocyclin produced a lower prevalence of salivary gland infections that was highly significant (2.3% infection, p<0.0001) compared to wild type and there were at least 10 times fewer trypanosomes in the glands. These established less intimate contact with gland tissue, and in the rare instances that parasites adhered to microvilli, they did not appear to attach tightly to epithelial cell surface structures (Fig 7C–F). The lower prevalence of mature infections by Δprocyclin was not due to a decrease in midgut infections after 4 weeks (no significant difference in total midgut infection prevalence), and is reflected by a substantially lower TI of 4.6%. Sequential transmissions of Δprocyclin through tsetse did not increase the prevalence or intensity of salivary gland infections, which could have been the case if a sub-population had acquired additional mutations or otherwise adapted itself to the lack of procyclins (data not shown).

To ascertain if there was a link between EP procyclin expression and colonisation of the salivary glands, infections were performed with Δproc/EP2+. Compared with Δprocyclin, however, the prevalence of salivary gland infections increased only marginally, to 3.9% infection (TI = 7.5%), and there was no obvious increase in the intensity of infection. One possible interpretation of these results is that trypanosomes might require more, or different procyclins in order to penetrate the proventriculus and gain access to the salivary glands. We can conclude, however, that procyclins are not essential for full cyclical transmission by the tsetse fly.

It could be hypothesised that multiple resistance genes carried by the null mutant and the addback might contribute to the reduction in maturation rates and/or the intensity of salivary gland infections. Although we felt this to be unlikely, given that the parasites used in transmission experiments were not exposed to antibiotics, we performed a control experiment with a null mutant for procyclin-associated genes (allPAGko) [25] that carries the identical set of resistance genes inserted downstream of the procyclin genes. In contrast to Δprocyclin, however, there was no significant difference in the prevalence and transmission index of this mutant compared to wild type AnTat 1.1, and the majority of salivary gland infections were of heavy or intermediate intensity (Figure 6B).

## Discussion

The sequential deletion of pairs of procyclin genes from a fly-transmissible strain of *T. brucei* resulted in the unexpected finding that the null mutant, Δprocyclin, was still able to complete the entire life cycle in tsetse and mammals. Δprocyclin was able to establish heavy infections in the fly midgut at approximately two-thirds of the prevalence of its wild type parent and was able to produce mature salivary gland infections and be transmitted by flies to mice. In addition, bloodstream forms of the mutant were capable of differentiating to the procyclic form without a detectable growth phenotype.

In this study we have formally proven for the first time that procyclins are not essential for the establishment of an infection in the ectoperitrophic space. The null mutant expresses free GPIs, however, and these may take over the function(s) of procyclins [20], [27]. The observation that Δprocyclin is notably more robust than other procyclin mutants that we have generated previously is most probably because of the experimental strategy that was used. It was necessary to do this because prolonged passage in rodents or in culture tends to select for parasites that lose the ability to differentiate and/or complete the life cycle. Deletion mutants were therefore subjected to fly transmission after each round of transfection, selecting for parasites that could adapt to a progressively thinner protein coat. This is in contrast to the EP/GPEET null mutant generated in bloodstream forms, which makes the transition from a full VSG coat to no glycoprotein coat when the parasite differentiates to the procyclic form [20] or to the Nour 6C mutant [21], which first becomes negative for procyclins when GPEET is repressed a few days post infection in the fly midgut [16].

The expression of GPEET mRNA is elaborately regulated by its 3′ UTR, both in the fly and in response to carbohydrate sources and mitochondrial activity in culture [16], [28]. In a wild type infection, down-regulation of GPEET correlates with the time that the trypanosome crosses the peritrophic matrix [16]. Nevertheless, trypanosomes lacking GPEET were able to establish midgut infections as efficiently as the wild type, in both teneral and non-teneral *G. m. morsitans*, even when a low infective dose was used. They were also equally competitive in mixed infections with the CFP-tagged wild type, which has the full complement of procyclins. The function of GPEET may only become apparent in other species of *Glossina* or under less favourable conditions for transmission. We have found that a mutant that constitutively expresses GPEET is transmitted very efficiently (unpublished data). The GPEET expressed by late procyclic forms and epimastigote forms was not phosphorylated, however, which is consistent with reports that the activity of the kinase is restricted to early procyclic forms [29].

On its own, the procyclin null mutant could colonise the midgut at rates and intensities comparable to the parental strain, and its reduced fitness only became apparent in mixed infections. This contrasts with a deletion mutant lacking all procyclin-associated genes, which could compete as effectively as the wild type in mixed infections [25]. There are two possible interpretations of these results. Midgut infections are reported to plateau at a constant number of ∼2×10^5^ parasites [30], [31]. The simplest explanation is that a fitter strain would replicate faster and occupy the available space before Δprocyclin would be able to do so. This implies that a coat of free GPIs cannot completely substitute for procyclins and may leave other surface molecules vulnerable to attack by proteases. Another possibility is that trypanosomes expressing procyclins might elicit an anti-microbial response that affects parasites that are not covered by the same coat. What the nature of this response might be is unknown at present, but it does not seem to involve an increase in defensin or attacin transcripts (M. Oberle, unpublished data).

The null mutant was able to produce mature infections, demonstrating that procyclins are not required to direct the trypanosomes to the salivary glands. The prevalence of salivary gland infections and transmission index were about 10-fold lower than with the wild type, however, and the number of trypanosomes was also reduced by more than one order of magnitude. Since procyclins are not normally detected on the surface of epimastigote forms [18], the lack of procyclins or the expression of free GPIs on the surface of midgut forms [20] might exert an indirect effect. Perturbation of the surface architecture of the parasite, for example by exposing surface molecules that are normally shrouded by procyclins, might alter interactions with host molecules. This, in turn, could conceivably reduce migration and/or adherence of epimastigote forms to the salivary gland epithelia. These hypotheses are extremely difficult to test, however, given the paucity of differentiation markers and the very low numbers of epimastigote forms in glands infected with Δprocyclin. The finding that the maturation rate could not be restored by the reintroduction of a single procyclin gene into the null mutant might be due to a requirement for multiple procyclin isoforms, in order for the parasite to migrate efficiently, or for appropriately regulated expression. Another possibility is that trypanosomes that have abundant free GPIs on their surface may have difficulty readjusting to attaching GPI anchors to proteins such as BARP, which might be needed for successful colonisation of the salivary glands. In line with this, the sequential deletion of procyclin genes resulted in progressively worse maturation rates and transmission indices (Figure 6A).

In conclusion, although procyclins are not essential, trypanosomes that express them are clearly at a competitive advantage and would probably eradicate procyclin-negative cells within one cycle of transmission. It is conceivable that a primordial trypanosome might originally have been covered by a glycocalyx of free GPIs and only later have acquired surface protein moieties that enhanced its survival and transmission. Intriguingly, a protein containing an extended EP repeat, tsetseEP, is also expressed in the midgut of tsetse flies [32] although the differences in codon usage makes it improbable that the parasite recently acquired this gene by horizontal transmission.

## Materials and Methods

### Trypanosomes

*T. b. brucei* AnTat 1.1 [33], and derivatives thereof, were used in this study. Bloodstream forms were cultured in HMI-9 containing 1.1% methylcellulose and supplemented with 10% fetal bovine serum (FBS) [34] or harvested from the blood of female NMRI mice or Wistar rats (Charles River Laboratories, France) that were immunosuppressed with cyclophosphamide (200 mg/kg body weight). Short stumpy bloodstream forms were harvested from mice 5–6 days post infection. Procyclic forms were cultured in SDM-79 [35] supplemented with 10% FBS and 20 mM glycerol. Bloodstream forms were triggered to differentiate to procyclic forms by transferring them to SDM-79 containing 20 mM glycerol, adding 6 mM *cis*-aconitate to the culture medium and lowering the incubation temperature to 27°C [8].

### Constructs, deletion and addback mutants

The constructs pCorleone-hyg, pCorleone-neo [20], pKOP, conferring resistance to bleomycin [21], and pKO-PAC, conferring resistance to puromycin [20], have been described previously. These were used sequentially to generate pairs of procyclin genes and replace them by an antibiotic resistance gene. The bicistronic construct pCorleone-EP2ΔLII-blast is based on pCorleone-CAT/GPEET [16], but in this case the first open reading frame encodes EP2 procyclin (previously EPβ, [21]) and the intergenic region between both open reading frames is derived from pGAPRONE [36]. The blasticidin resistance gene was amplified from pHD 887 [37] (courtesy of C. Clayton, Heidelberg) using the primer pair 5′-GCTAGCTAGCATGGCCAAGCCT-3′ and 5′-CCATCGATACTCACAGCGACTA-3′ and cloned between the *NheI* and *ClaI* sites from the newly derived construct, thereby replacing the phleomycin-resistance gene. Stable transformation of bloodstream forms and procyclic forms were performed as described [16], [34]. In each case, clones were analysed for correct integration of the construct before being transmitted through tsetse and mice. The resulting bloodstream forms were triggered to differentiate to procyclic forms in culture and DNA was isolated and reanalysed before the next knockout was performed.

### Infection of tsetse flies

Pupae of *Glossina morsitans morsitans* were obtained from the International Atomic Agency (Vienna), the Department of Entomology, Slovak Academy of Science (Bratislava) or the Institute of Tropical Medicine, Antwerp. Teneral flies were infected with trypanosomes during the first blood meal as described [21]. Non-teneral flies were infected with the second blood meal after emergence. The blood meal consisted either of 2×10^6^ short stumpy bloodstream forms in defibrinated horse blood or, alternatively, of 2×10^6^ procyclic forms in SDM-79 supplemented with washed horse red blood cells. Infected flies were fed three blood meals per week through artificial membranes. Flies were analyzed for midgut infections by dissection of their midguts and for mature infections by the presence of metacyclic forms in saliva from flies extruded onto slides. Midgut infections were scored quantitatively as light, intermediate or heavy by criteria defined by Ruepp et al. [21]. To complete cyclical transmission, flies were fed on anesthetized NMRI mice, which were subsequently monitored for parasitaemia. The two tailed Fisher's exact test was used for all statistical analyses (Daan G Uitenbroek, “SISA-Binomial.” 1997; 〈http://www.quantitativeskills.com/sisa/distributions/binomial.htm〉).

### Immunofluorescence and transmission electron microscopy

Immunofluorescence. Procyclic culture forms were fixed with 2% formaldehyde at 4°C. Rabbit polyclonal anti-GPEET antiserum (K1; [21], [38] was used at a dilution of 1∶500. The monoclonal antibody TRBP1/346, which recognises the first 20 amino acids of EP procyclins [39], was diluted 1∶1000. Alexa 488-conjugated anti-rabbit (Molecular Probes) and TRITC-conjugated anti-mouse (Sigma) secondary antibodies were used at 1∶500.

For transmission electron microscopy, salivary glands were isolated from infected flies and fixed for 4 hrs at 4°C in a solution containing 0.1 M cacodylate buffer, pH 7.4 and 2% glutaraldehyde. Subsequent treatments were performed exactly as described [21]. All preparations were observed using a transmission electron microscope (model 600, Philips Technologies, Cheshire, CT) operating at 60 kV.

### Ethanolamine labelling

Early procyclic forms (cultured in SDM-79 supplemented with 20 mM glycerol) or late procyclic forms (cultured in SDM-79 without glycerol) were labelled with [^3^H]ethanolamine for 18 h as described [20].The delipidated pellets were extracted with CMW to solubilise free GPIs, followed by 9% butan-1-ol (but) and 0.1% Triton X-100 (TX) to solubilise procyclins. The CMW extracts were dried and partitioned between water and butan-1-ol; the aqueous phase contains free GPIs (CMWaq). All fractions were analyzed by SDS-PAGE and fluorography.

### Competition experiments

CFP#5, a clone of AnTat 1.1 tagged with cyan fluorescent protein [25] was used as a reference in co-culture experiments and mixed infections. Stumpy bloodstream forms of CFP#5 were mixed 1∶1 with either wild type AnTat1.1, ΔGPEET or Δprocyclin and triggered to differentiate in culture or used to infect tsetse as described above. Procyclic forms were used for mixed infections as described previously [25]. Midgut forms were isolated from infected flies and fixed with 4% formaldehyde in order to preserve fluorescence. The percentage of CFP-positive cells (pooled from 5–10 infected flies) was determined by fluorescence microscopy.

## Supporting Information

Figure S1 Southern blot analysis of deletion mutants
(0.17 MB TIF)
