# Supplementary figures and images for: Major Surface Glycoproteins of Insect Forms of Trypanosoma brucei Are Not Essential for Cyclical Transmission by Tsetse
Source: PLoS One. 2009 Feb 18;4(2):e4493. doi: 10.1371/journal.pone.0004493 (PMC2637416; doi:10.1371/journal.pone.0004493)

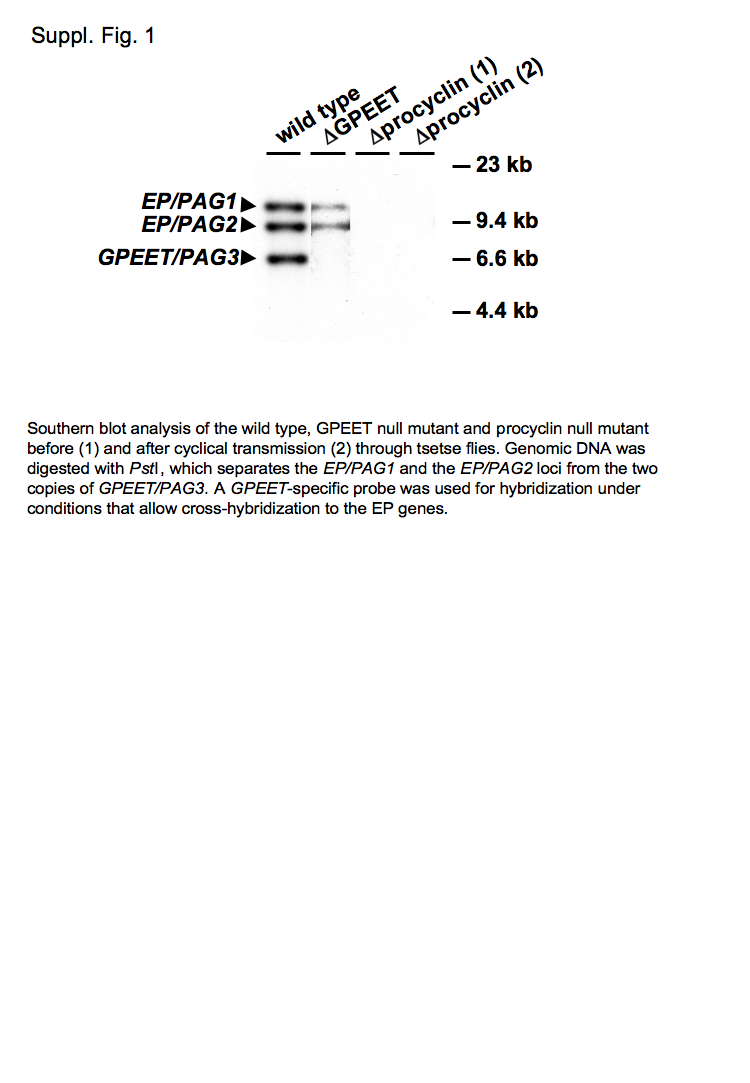

Supplement: Figure S1 — Southern blot analysis of deletion mutants (0.17 MB TIF) [file pone.0004493.s001.tif]
